# Prion protein amino acid sequence influences formation of authentic synthetic PrP^Sc^

**DOI:** 10.1038/s41598-022-26300-0

**Published:** 2023-01-09

**Authors:** Alyssa J. Block, Taylor C. York, Romilly Benedict, Jiyan Ma, Jason C. Bartz

**Affiliations:** 1 Department of Medical Microbiology and Immunology, School of Medicine, Creighton University, 2500 California Plaza, Omaha, NE 68178 USA; 2 Department of Plant, Soil, and Microbial Sciences, College of Agriculture and Natural Resources, Michigan State University, East Lansing, MI USA; 3 Van Andel Institute, Center for Neurodegenerative Science, Grand Rapids, MI USA

**Keywords:** Pathogens, Prions

## Abstract

Synthetic prions, generated de novo from minimal, non-infectious components, cause *bona fide* prion disease in animals. Transmission of synthetic prions to hosts expressing syngeneic PrP^C^ results in extended, variable incubation periods and incomplete attack rates. In contrast, murine synthetic prions (MSP) generated via PMCA with minimal cofactors readily infected mice and hamsters and rapidly adapted to both species. To investigate if hamster synthetic prions (HSP) generated under the same conditions as the MSP are also highly infectious, we inoculated hamsters with HSP generated with either hamster wild type or mutant (ΔG54, ΔG54/M139I, M139I/I205M) recombinant PrP. None of the inoculated hamsters developed clinical signs of prion disease, however, brain homogenate from HSP^WT^- and HSP^ΔG54^-infected hamsters contained PrP^Sc^, indicating subclinical infection. Serial passage in hamsters resulted in clinical disease at second passage accompanied by changes in incubation period and PrP^Sc^ conformational stability between second and third passage. These data suggest the HSP, in contrast to the MSP, are not comprised of PrP^Sc^, and instead generate authentic PrP^Sc^ via deformed templating. Differences in infectivity between the MSP and HSP suggest that, under similar generation conditions, the amino acid sequence of PrP influences generation of authentic PrP^Sc^.

## Introduction

Prion diseases are neurodegenerative disorders that are inevitably fatal and can be transmissible. Prions are comprised of PrP^Sc^, the disease-specific conformation of the host-encoded prion protein, PrP^C^. Formation of PrP^Sc^ occurs when PrP^C^ binds to PrP^Sc^ and, through a largely unknown process, PrP^Sc^ directs a complete restructuring of PrP^C^ from a monomeric, α-helical-rich conformation to a multimeric, fibrillar parallel in-register intermolecular β-sheet/stack (PIRIBS) conformation^1–7^. Prion diseases affect numerous species, including humans, and are clinically characterized by cognitive and/or motor deficits and neuropathologically characterized by spongiform degeneration, reactive astrocytosis and lack of an inflammatory response.

Prions have zoonotic potential. Transmission of prions to a new species can result in an extension of the incubation period and a reduction in attack rate (e.g., species barrier effects) compared to intraspecies transmission in the original host species^8^. Subsequent serial intraspecies transmission can result in a shortening of the incubation period and stabilization of the disease phenotype^9–13^. Alternatively, interspecies transmission can result in failure of the prion to adapt to the new species, instead retaining pathogenicity for the original host species. The species barrier is dictated by compatibility of the conformations of PrP^Sc^ and PrP^C^. This can be influenced by strain-specific differences in the conformation of PrP^Sc^ and by primary amino acid sequence differences between the agent PrP^Sc^ and host PrP^C^. The exact mechanism(s) underlying these observations, however, is unknown.

Prions can be generated from noninfectious components. Protein misfolding cyclic amplification (PMCA) can generate synthetic prions from minimal components such as PrP^C^, RNA and lipids that recapitulate several aspects of brain-derived prions^14–16^. Synthetic prions possess a C-terminal, protease-resistant core, and can be serially propagated in PMCA and cell culture^14–18^. Inoculation of synthetic prions into animals expressing syngeneic PrP results in development of clinical signs of prion disease and neuropathological hallmarks of prion disease that include spongiform degeneration, reactive gliosis and deposition of PrP^Sc^^14,15,17–22^. Intraspecies transmission of synthetic prions often results in a long and highly variable incubation period and an incomplete attack rate^14,17,18,20,21,23^. These species barrier-like effects observed with synthetic prions are proposed to be a result of deformed templating, a model of prion conversion which posits synthetic prions do not have the same conformation as authentic PrP^Sc^. Instead, synthetic prions are hypothesized to be comprised of a fibrillar PrP conformation that, through an inefficient process of generating folding intermediates, results in atypical PK-resistant PrP (i.e., PrP^res^) prior to production of authentic PrP^Sc^^24,25^. An exception to this is the murine synthetic prions (MSP) produced under PMCA conditions that are highly infectious for mice and can efficiently cross the species barrier to hamsters, suggesting these MSPs are bona fide PrP^Sc^^15,26^. Here, we investigated whether hamster synthetic prions, created using the same process as the highly infectious MSPs, were infectious for hamsters.

## Results

### Transmission of hamster synthetic prions to hamsters

**Figure 1:**
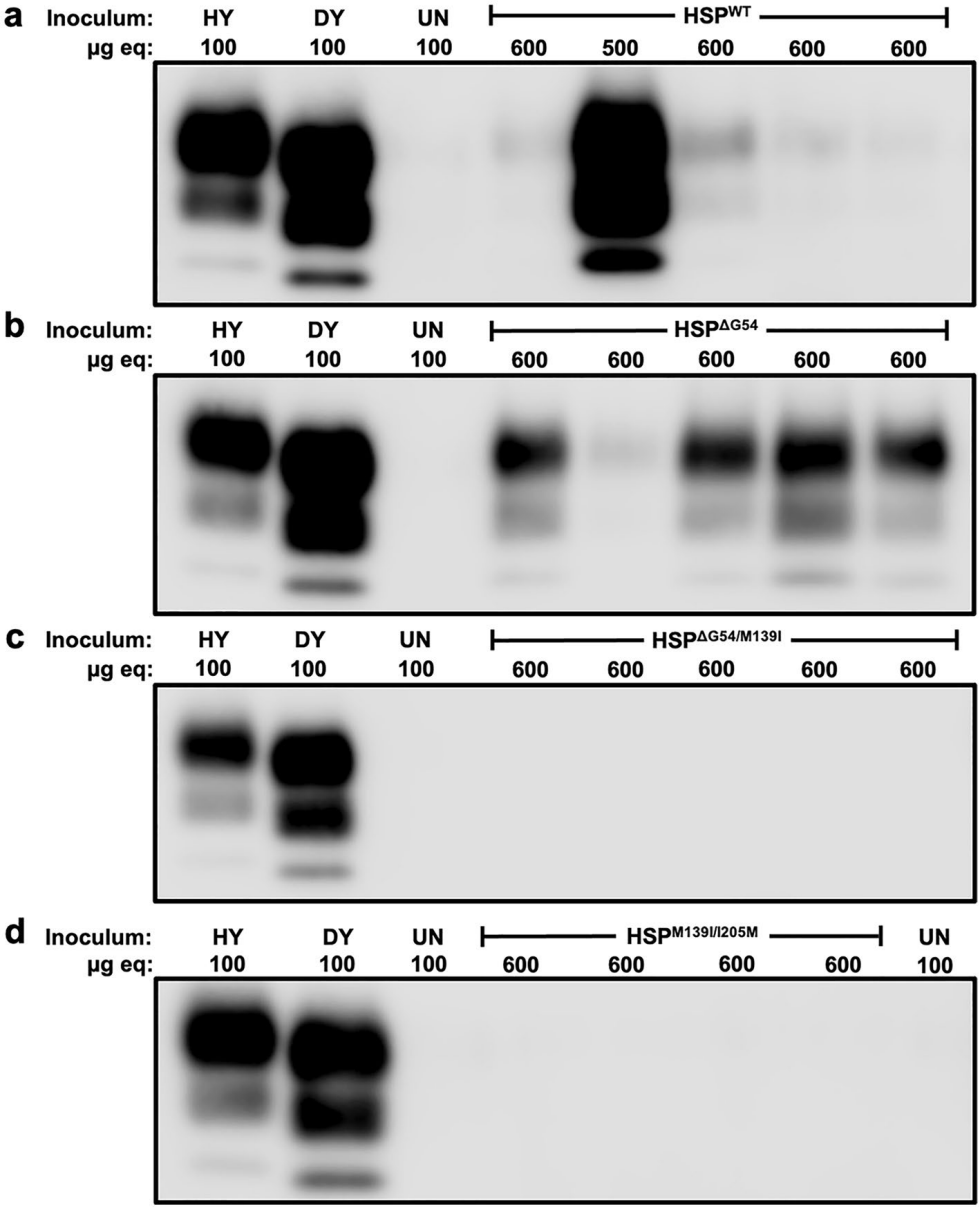
Evidence of subclinical prion infection in hamsters inoculated with HSP^WT^ or HSP^ΔG54^. Representative Western blots of brain homogenate from (**a**) HSP^WT^-, (**b**) HSP^ΔG54^-, (**c**) HSP^ΔG54/M139I^-, or (**d**) HSP^M139I/I205M^-infected hamsters. Western blot analysis with the anti-PrP antibody 3F4 identified PrP^Sc^ in brain homogenate from HSP^WT^- and HSP^ΔG54^-infected hamsters and failed to identify PrP^Sc^ in brain homogenate from HSP^ΔG54/M139I^- or HSP^M139I/I205M^-infected hamsters. Due to an intercurrent death at 303 dpi, the group inoculated with HSP^M139I/I205M^ has only four animals. Microgram equivalents (µg eq) loaded for each lane are listed above the blot. This experiment was repeated at least three times with similar results. Blots were cropped to focus on PrP^Sc^. The original, uncropped blots are in Supplemental Fig. S3.

Groups of 3–4-week-old hamsters (n = 5 per group) were intracerebrally (i.c.) inoculated with either uninfected brain homogenate (UN), hamster wild type synthetic prions (HSP^WT^), or hamster mutant synthetic prions (HSP^ΔG54^, HSP^ΔG54/M139I^, or HSP^M139I/I205M^) (Supplemental Figs. S1, S2). All hamsters i.c. inoculated with UN brain homogenate, HSP^WT^, HSP^ΔG54^, HSP^ΔG54/M139I^, or HSP^M139I/I205M^ remained clinically normal for greater than 500 days post infection (Table 1). There was one intercurrent death at 303 dpi in the group inoculated with HSP^M139I/I205M^. Western blot analysis of proteinase K (PK)-digested brain homogenate from hamsters inoculated with HSP (WT or mutants) identified low levels of PrP^Sc^ in the CNS from all (n = 5) HSP^WT^- and HSP^ΔG54^-infected hamsters compared to hamsters infected with brain-derived prion strains HY and DY TME (Fig. 1, Supplemental Fig. S2), indicating a subclinical prion infection. Western blot analysis failed to detect PK-resistant PrP^Sc^ in HSP^ΔG54/M139I^- or HSP^M139I/I205M^-infected hamsters (Fig. 1, Supplemental Fig. S2).

**Table 1:** Transmission and adaptation of hamster synthetic prions to hamsters.

Inoculum	Incubation period	Clinical duration	Avg. Weight (grams)^a^	PrP^Sc^ Detected by WB^b^
**Synthetic prions**				
Mouse WT	321 ± 3^c^ (5/5)^d^	66 ± 3	n.d.^e^	5/5^f^
Hamster WT	$\ge$ 500 (0/5)	n.a.^g^	n.a	5/5
Hamster ΔG54	$\ge$ 500 (0/5)	n.a	n.a	5/5
Hamster ΔG54/M139I	$\ge$ 500 (0/5)	n.a	n.a	0/5
Hamster M139I/I205M	$\ge$ 500 (0/5)^h^	n.a	n.a	0/5
Uninfected b.h.^i^	$\ge$ 500 (0/5)	n.a	n.a	0/5
**Brain derived prions**				
1st ha. pass. HSP^WT^ b.h.	335 ± 6 (5/5)	41 ± 3	200	5/5
1st ha. pass. HSP^ΔG54^ b.h.	305 ± 5 (5/5)	60 ± 2	196 ± 8^j^	5/5
Uninfected b.h.	$\ge$ 400 (0/5)	n.a	148^k^	0/5
2nd ha. pass. HSP^WT^ s.c.h.^l^	168 ± 3 (5/5)	57 ± 3	198 ± 7	5/5
2nd ha. pass. HSP^ΔG54^ s.c.h.	315 ± 10 (5/5)	81 ± 10	214 ± 2	5/5
Uninfected b.h.	$\ge$ 430 (0/5)	n.a	170-175^m^	0/5

^a^At onset of clinical signs.

^b^WB—Western blot.

^c^Days ± SEM.

^d^Number affected/number inoculated.

^e^n.d.—no data.

^f^PrP^Sc^ detected/number inoculated.

^g^n.a.—not applicable.

^h^One intercurrent death at 303 dpi.

^i^b.h.—brain homogenate.

^j^Average weight in grams ± SEM.

^k^Average weight in grams at HSP clinical onset.

^l^s.c.h.—spinal cord homogenate.

^m^Average weight in grams range at HSP clinical onset.

### Emergence of clinical infection and slow adaptation of HSP to hamsters

**Figure 2:**
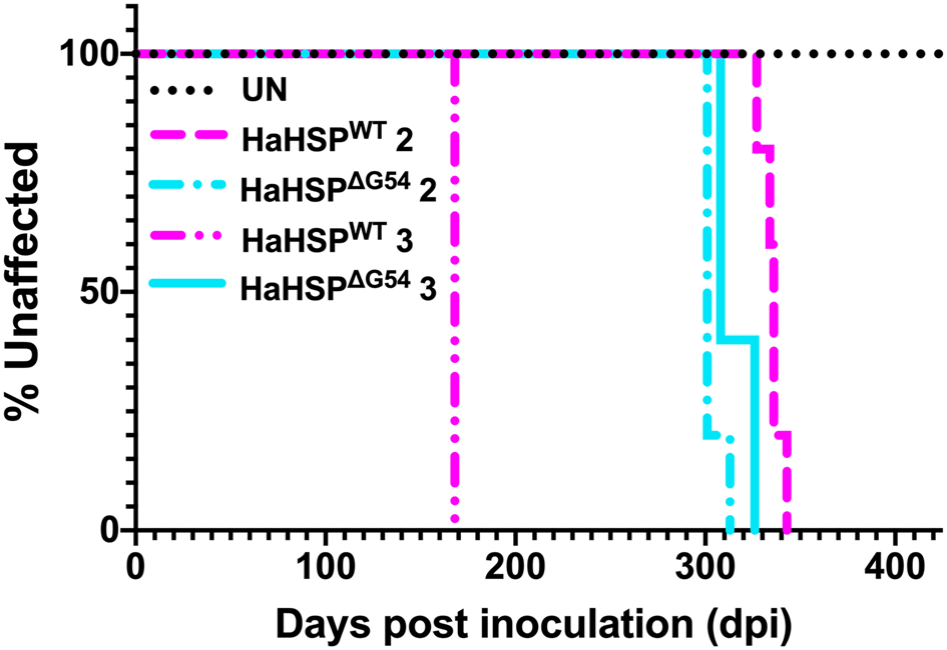
Incubation periods of HaHSP^WT^- and HaHSP^ΔG54^-infected hamsters diverge during serial passage. Survival curves depicting the changes in the incubation periods of HaHSP^WT^- and HaHSP^ΔG54^-infected hamsters during adaptation. The incubation periods of both HaHSP^WT^- and HaHSP^ΔG54^-infected hamsters at second passage were prolonged (335 ± 6 and 305 ± 5 dpi, respectively). However, at third passage, the incubation period of HaHSP^WT^-infected hamsters shortened to 168 ± 3 dpi. In contrast, the incubation period of HaHSP^ΔG54^-infected hamsters at third passage remained relatively stable (315 ± 10 dpi). This divergence of incubation periods at third passage corresponds to divergence in clinical signs as well.

CNS material from hamsters subclinically infected with HSP^WT^ or HSP^ΔG54^ was serially passaged twice in hamsters. Groups of hamsters (n = 5 per group) were i.c. inoculated with UN, HSP^WT^-, or HSP^ΔG54^-infected brain (2nd passage) or spinal cord (3^rd^ passage) homogenate. All (n = 5) hamsters inoculated with either hamster passaged HSP^WT^ (HaHSP^WT^) or HSP^ΔG54^ (HaHSP^ΔG54^) at both serial passages developed clinical signs of prion infection with incubation periods of 335 ± 6 and 305 ± 5 dpi at second passage and 168 ± 3 dpi and 315 ± 10 dpi at third passage, respectively (Fig. 2, Table 1). Hamsters inoculated with UN brain homogenate remained clinically normal for more than 400 dpi for both serial passages. Clinical disease progression was prolonged, with a clinical phase of 41 ± 3 and 60 ± 2 days at second passage and 57 ± 3 and 81 ± 10 days at third passage for HaHSP^WT^- and HaHSP^ΔG54^-infected hamsters, respectively (Table 1). At second passage, HaHSP^WT^- and HaHSP^ΔG54^-infected hamsters were characterized clinically by ataxia, lethargy, and progressive weight gain (Table 1). However, one animal inoculated with HaHSP^ΔG54^ presented with hyperexcitability and lacked the progressive weight gain observed in the other HaHSP^ΔG54^-infected animals. By the third serial hamster passage, clinical signs of HaHSP^WT^-infected hamsters included hyperexcitability and a trembling that developed into ataxia. In contrast, HaHSP^ΔG54^-infected hamsters at third passage were clinically characterized by a mild hyperexcitability that developed into lethargy. Progressive weight gain remained a shared clinical characteristic of HaHSP^WT^- and HaHSP^ΔG54^-infected hamsters. Overall, clinical prion disease was established at second passage for both HaHSP^WT^- and HaHSP^ΔG54^-infected hamsters and HaHSP^WT^- and HaHSP^ΔG54^-infected hamsters were clinically similar until third passage.

### Electrophoretic mobility and glycoform ratio of PrP^Sc^ from HaHSP^WT^- and HaHSP^ΔG54^-infected hamsters

**Figure 3:**
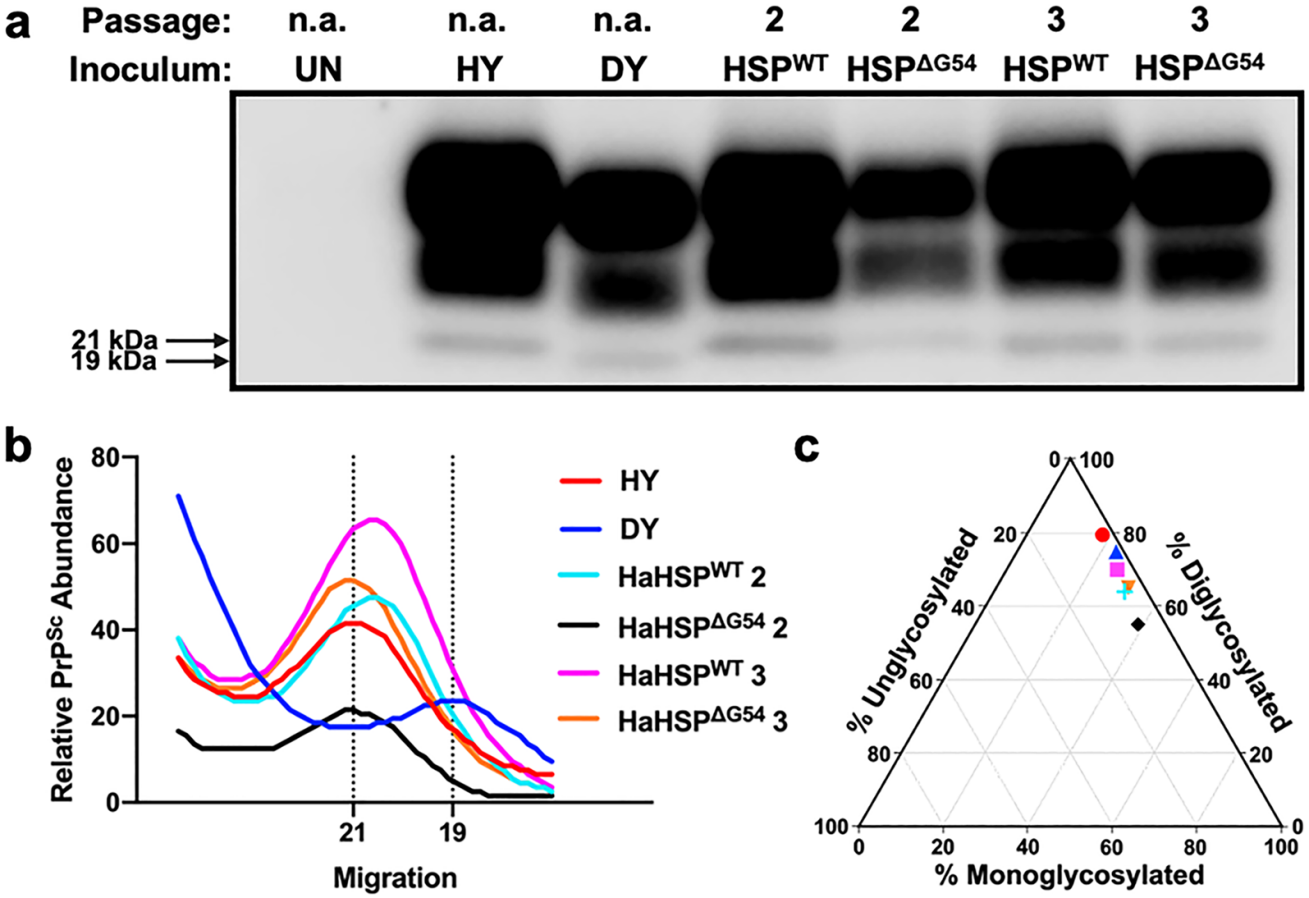
The electrophoretic mobility and glycoform ratio of PrP^Sc^ from HaHSP^WT^- and HaHSP^ΔG54^-infected hamsters are consistent with brain-derived strains. Western blot (**a**), migration analysis (**b**), and glycoform ratio (**c**) of PrP^Sc^ from the CNS of hamsters infected with HY, DY, or second or third hamster passage of hamster synthetic prions (HaHSP^WT^ or HaHSP^ΔG54^). The unglycosylated polypeptide of PrP^Sc^ from HaHSP^WT^- or HaHSP^ΔG54^-infected CNS homogenate at either second or third passage migrates at 21 kDa, similar to PrP^Sc^ from HY-infected CNS homogenate and dissimilar to PrP^Sc^ from DY-infected CNS homogenate, which migrates at 19 kDa. The glycoform ratios of PrP^Sc^ from HY-, DY-, HaHSP^WT^- or HaHSP^ΔG54^-infected CNS homogenate is similar, with diglycosylated PrP^Sc^ as the dominant glycoform. This experiment was repeated at least three times with similar results. The blot in panel a was cropped to focus on PrP^Sc^. The original, uncropped blot is in Supplemental Fig. S4.

Western blot analysis of PK-digested spinal cord homogenate (SCH) from HaHSP^WT^- and HaHSP^ΔG54^-infected hamsters identified PK-resistant PrP^Sc^, consistent with prion infection in all clinically positive animals from second and third passage (Fig. 3A). The unglycosylated PrP^Sc^ polypeptide from HaHSP^WT^- and HaHSP^ΔG54^-infected brain homogenate migrates at 21 kDa, consistent with all known hamster strains except DY, which migrates at 19 kDa (Fig. 3B)^27,28^. Glycoform analysis of SCH from second and third passage of HaHSP^WT^ and HaHSP^ΔG54^ in hamsters indicates the diglycosylated glycoform is the most abundant glycoform, consistent with all known hamster strains^29,30^ (Fig. 3C). Overall, the migration and glycoform ratio of PrP^Sc^ from HaHSP^WT^- and HaHSP^ΔG54^-infected hamsters remained consistent during serial passage and did not differ from other hamster-adapted prion strains.

### The conformational stability of PrP^Sc^ from HaHSP^WT^- and HaHSP^ΔG54^-infected hamsters changes during serial passage in hamsters

**Figure 4:**
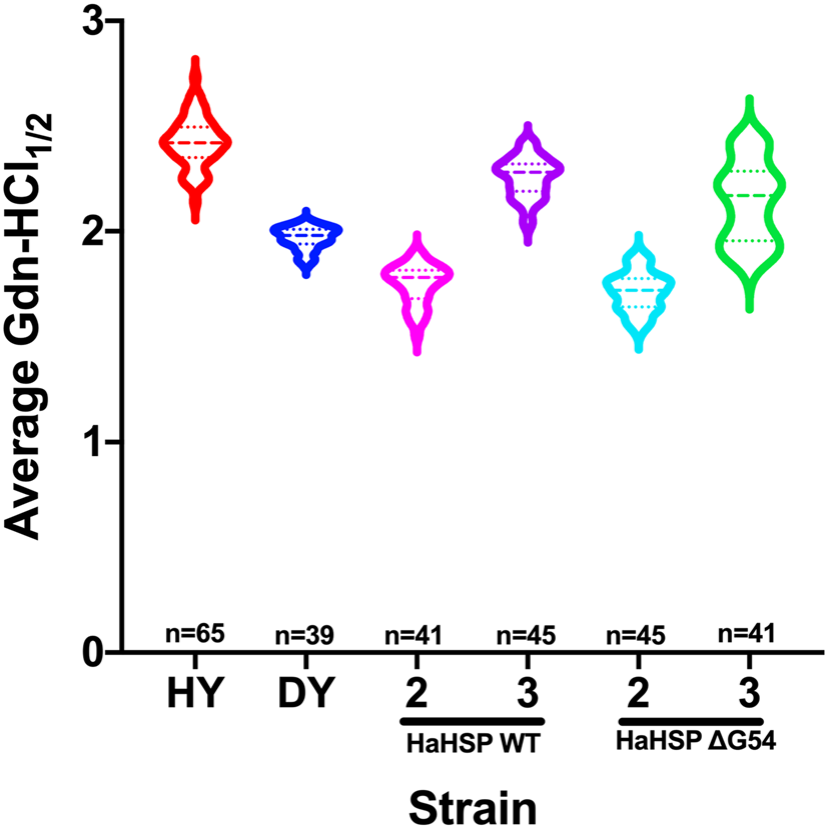
Conformational stability of PrP^Sc^ from hamsters infected with HaHSPs changes during serial passage. Conformational stability of PrP^Sc^ from hamsters infected with either hyper (HY), drowsy (DY), hamster passage two and three of hamster WT synthetic prions (HaHSP WT), or hamster passage two and three of hamster Δ54 synthetic prions (HaHSP ΔG54) represented as a violin plot. ANOVA analysis determined the stability of PrP^Sc^ from HY-, DY-, second and third passage HaHSP^WT^- and HaHSP^ΔG54^-infected brain homogenate all significantly (*p* < 0.05) differed except for PrP^Sc^ from the second passages of HaHSP^WT^ and HaHSP^ΔG54^ in hamsters which did not significantly (*p* > 0.05) differ. This indicates stability changed during serial passage and is consistent with HaHSP^WT^- and HaHSP^ΔG54^-infected hamsters sharing similar clinical characteristics at second passage that diverged at third serial passage. The ‘2’ and ‘3’ on the x-axis indicates the passage number. The dashed line within each violin plot represents the median and the dotted lines represent the first and third quartile. n indicates the number of technical replicates per strain/passage. There were five animals per HaHSP strain and passage and 8–11 replicates per animal.

The average conformational stability [Gdn-HCl]_½_ of PrP^Sc^ from the CNS of hamsters infected with brain-derived control strains HY or DY TME was 2.42 ± 0.01 and 1.97 ± 0.01 M, respectively (Fig. 4). Conformational stability of PrP^Sc^ from the first passage of HSP^WT^ and HSP^Δ54^ in hamsters was not assessed due to low levels of PrP^Sc^ in the CNS of these animals. The average conformational stability [Gdn-HCl]_½_ of PrP^Sc^ from the CNS of HaHSP^WT^- and HaHSP^ΔG54^-infected hamsters at second passage was 1.75 ± 0.01 and 1.71 ± 0.01 M, respectively, significantly (*p* < 0.05) less stable than PrP^Sc^ from the CNS of HY- or DY-infected controls (Fig. 4). At third passage, the average conformational stability [Gdn-HCl]_½_ of PrP^Sc^ from the CNS of HaHSP^WT^- and HaHSP^ΔG54^-infected hamsters increased to 2.26 ± 0.01 and 2.14 ± 0.03 M, respectively, significantly (*p* < 0.05) more stable than PrP^Sc^ from the CNS of DY-infected hamsters but significantly (*p* < 0.05) less stable than PrP^Sc^ from the CNS of HY-infected hamsters (Fig. 4). The average conformational stability of PrP^Sc^ from both HaHSP^WT^- and HaHSP^ΔG54^-infected hamsters increased between second and third passage. This is in contrast to PrP^Sc^ from hamsters infected with murine synthetic prions, which remained stable throughout serial passage^26^. Overall, the conformational stability of PrP^Sc^ changed throughout serial passage and is intermediate between DY and HY controls.

### HaHSP^WT^- and HaHSP^ΔG54^-infected hamsters are characterized by the classical neuropathological hallmarks of prion disease

**Figure 5:**
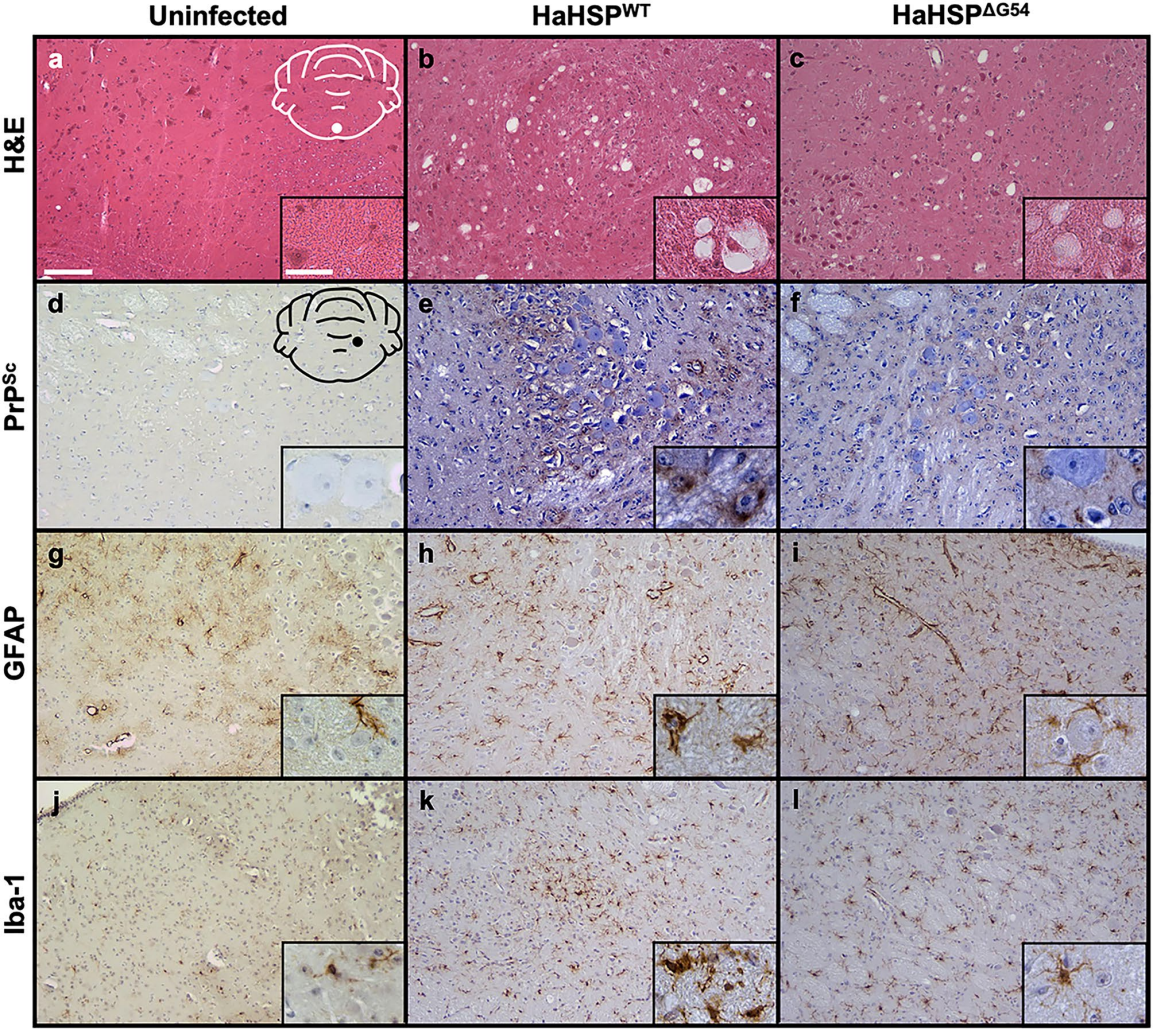
Brains of HaHSP^WT^- or HaHSP^ΔG54^ -infected hamsters are characterized by the histopathological hallmarks of prion disease. Brain sections from mock-infected (UN), second passage HaHSP^WT^-, and second passage HaHSP^ΔG54^-infected animals were stained with hematoxylin and eosin (**a**–**c**) to observe spongiform degeneration. Immunohistochemistry was also performed using the anti-PrP antibody 3F4 (**d**–**f**), the astrocyte marker GFAP (**g**–**i**), and the microglial marker Iba-1 (**j**–**l**) to observe abnormal PrP deposition, astrogliosis, and microgliosis, respectively. The white schematic inset in (**A)** depicts the brain region imaged in (**a**–**c**). The black schematic in (**d)** depicts the brain region imaged in (**d**–**l**). Scale bar 100 μm; inset scale bar 25 μm.

Hematoxylin and eosin staining of HaHSP^WT^- and HaHSP^ΔG54^-infected brain sections revealed characteristic spongiosis associated with prion disease (Fig. 5b, c) whereas brains from mock-infected animals lacked spongiosis (Fig. 5A). Immunohistochemistry (IHC) with the anti-PrP antibody 3F4 identified abnormal prion protein deposition in the brains of HaHSP^WT^- and HaHSP^ΔG54^-infected animals (Fig. 5E, F) but was not identified in mock-infected animals (Fig. 5D). Compared to brain sections from mock-infected animals (Fig. 5G, J), HaHSP^WT^- and HaHSP^ΔG54^-infected brain sections also showed astrogliosis (Fig. 5H, I) and microgliosis (Fig. 5K, L) when the astrocyte marker GFAP and microglia marker Iba-1 were utilized in IHC, respectively. Overall, HaHSP^WT^- and HaHSP^ΔG54^-infected hamsters exhibit the neuropathological hallmarks of prion disease, similar to animals infected with brain-derived prions.

## Discussion

Murine and hamster synthetic prions (MSP and HSP, respectively) produced under identical conditions have vastly different capacities for establishing prion disease and adapting to hamsters. Bacterially-generated murine or hamster recombinant PrP (recPrP) underwent serial PMCA in the presence of RNA and an endogenous lipid, POPG to produce MSP and HSP^15^. Interspecies transmission of the MSP to hamsters was more efficient than intraspecies transmission of other synthetic prions^14,17,18,20,24,26^. Additionally, the MSP rapidly adapted to hamsters and the biochemical characteristics of PrP^Sc^ from hamster-adapted MSP (HaMSP) remained stable throughout serial passage. These results were consistent with the intraspecies transmission of the MSP to mice, which resulted in a 100% attack rate and relatively short, stable incubation period at first passage^15^. Overall, the rapid adaptation of the MSP to both mice and hamsters and the stability of clinical and biochemical characteristics suggested the MSP are high titer, authentic PrP^Sc^ composed of a single strain. In stark contrast, in the current study, hamsters inoculated with HSP^WT^ failed to develop clinical signs of prion disease at first passage, but subclinical infection was indicated by the presence of PK-resistant PrP in brains of HSP^WT^-infected hamsters (Fig. 1).

HSP^WT^ adapted slowly to hamsters. Clinical onset occurred at second passage following an extended (335 ± 6 dpi) incubation period, which shortened (168 ± 3 dpi) by third passage. In contrast to the MSP, the conformational stability of PrP^Sc^ from HSP^WT^-infected hamsters increased as the incubation period decreased. Fluctuations in conformational stability during serial passage is observed with other synthetic prions as well^18,20,31^. Dynamic PrP^Sc^ conformational stability during serial passage suggests continual adaptation of HSP^WT^ to hamsters and selection of a dominant strain from a mixture. We hypothesize that the PMCA cofactors and conditions used to generate the MSP and HSP^WT^ favored formation of authentic PrP^Sc^ using murine recPrP but not hamster recPrP, with HSP^WT^ triggering formation of *bona fide* PrP^Sc^ through the process of deformed templating.

The deformed templating conversion model is consistent with the observed transmission properties of HSP to hamsters. Both SSLOW (Synthetic Strain Leading to OverWeight)-, the prototypic strain of the deformed templating model, and HSP^WT^-infected hamsters did not develop clinical signs of prion infection at first passage, instead developing clinical signs at second passage following an extended incubation period^18^. The conformational stability of PrP^Sc^ from SSLOW- and HSP^WT^-infected hamsters changed throughout adaptation, decreasing or increasing, respectively, as the incubation period shortened. Although SSLOW and HSP share similarities, they have markedly different neuropathology. The neuropathology of SSLOW-infected hamsters is characterized by large PrP^Sc^ deposits, which are notably absent in HSP neuropathology (Fig. 5)^18,32^. Transmission of HSP^WT^ and SSLOW to hamsters is similar, suggesting HSP^WT^ established prion disease via deformed templating. The serial seeded generation of the HSP could also be considered a deformed templating process. The MSP could not directly seed hamster WT recPrP, but instead could seed double mutant hamster recPrP. The double mutant synthetic prions then could seed single mutant hamster recPrP and the single mutant synthetic prions could seed hamster WT recPrP. We hypothesize this step-wise generation process of the HSP may produce synthetic prions at a different point in the deformed templating process (i.e., atypical PrP-res versus fibrillar amyloid). As SSLOW synthetic prions are hypothesized to be fibrillar amyloid, the HSP being further along in the deformed templating process could account for observed differences in establishment of infection and adaptation between the two synthetic prions. Additionally, differences between SSLOW and HSP could result from differences in generation conditions, with SSLOW synthetic prions formed de novo under denaturing and shaking conditions whereas HSP formed using PMCA. PMCA can expedite interspecies transmission and adaptation of brain-derived prions and thus could expedite the deformed templating process^33^. We cannot exclude the possibility that HSP contains low titer PrP^Sc^, however, we think this is unlikely as we would expect PMCA to generate higher titer material of a single strain similar to what we observed with MSP^26^. Overall, despite differences in generation of and disease phenotype caused by the HSP and SSLOW, both utilize a similar conversion pathway to establish infection.

Structural differences between synthetic prions and brain-derived PrP^Sc^ could explain the need for synthetic prions to utilize deformed templating to generate authentic PrP^Sc^. Recent cryo-EM studies have revealed the structures of both synthetic prion fibrils formed from human recombinant PrP and infectious PrP^Sc^ derived from patient samples^34–37^. The monomeric structures of human synthetic prion fibrils and brain-derived PrP^Sc^ from GSS patients differed, as did interfacing of the monomers within the protofilaments comprising the prion fibrils^34–37^. This could explain the observed differences in infectivity, in which the synthetic fibril structure is less efficient at PrP^Sc^ conversion. This incongruity has also been observed in α-synuclein. Inoculation of brain homogenate from MSA patients to TgM83^+/−^ mice results in an average incubation of ~ 120 dpi whereas inoculation of preformed fibrils to TgM83^+/−^ mice results in highly variable incubation periods ranging from ~ 90 to 330 dpi^38–40^. The differences in incubation period correspond to differences in structure between the preformed fibrils and patient-derived α-synuclein^41–43^. Overall, the structural disparity between in vitro generated fibrils and brain-derived prions is a possible explanation for the inefficiency of synthetic prions following intraspecies transmission and supports the role of deformed templating in establishing infection.

Mutations in the hamster recPrP used to generate the HSP affected infectivity and strain emergence. Amino acid sequence differences between murine and hamster PrP at residues 54, 139, and 205 greatly impact the mouse/hamster species barrier. In the current study, the mutations introduced to hamster PrP (ΔG54, ΔG54/M139I, and M139I/I205M) increased the similarity of the hamster PrP amino acid sequence to the murine PrP sequence. Of the three HSPs generated using mutant hamster recPrP, only the HSP^ΔG54^ mutant caused subclinical infection, whereas the other mutants, HSP^ΔG54/M139I^ and HSP^M139I/I205M^, failed to establish infection in hamsters. Interestingly, HSP^ΔG54/M139I^ and HSP^M139I/I205M^ share a substitution of the hamster methionine at residue 139 for the murine isoleucine (Supplemental Fig. S1). Studies in Sc^+^-MNB cells, cell-free conversion systems, and transgenic mice have found that methionine or isoleucine expressed at residue 138/139 (murine/hamster numbering) confers resistance or susceptibility to prion infection^44–46^. This susceptibility or resistance depends on which PrP background a mutation is introduced (mouse or hamster) and the strain of PrP^Sc^ used to test conversion^44–46^. Recent cryo-EM data comparing the structure of anchorless RML mouse and 263 K hamster PrP^Sc^ highlights the structural differences between these strains at residue 138/139^1,47^. The substitution of isoleucine at residue 139 in hamster recombinant PrP may affect the structure of the resulting HSP, subsequently affecting its ability to seed conversion of hamster WT PrP^C^. Taken together, these studies indicate residue 139 plays an important role in the mouse/hamster species barrier and mutation at this residue may hinder conversion of hamster PrP^C^ to PrP^Sc^ via deformed templating.

## Materials and methods

### Ethics statement

All procedures involving animals were approved by the Creighton University Institutional Animal Care and Use Committee and comply with the *Guide for the Care and Use of Laboratory Animals* and ARRIVE guidelines*.*

### Synthetic prions

The murine synthetic prions (MSP) were generated as previously described^15,19,26,48^. Briefly, murine recombinant PrP (PrP23-230) was expressed in E. coli and purified^48^. Murine recombinant PrP (25 μg/ml in deionized H2O), 1-palmitoyl-2-oleoylphophatidylglycerol (POPG; 22.2 μg/ml in 20 mM Tris HCl, pH 7.4), and total RNA isolated from mouse liver (150 μg/ml) were mixed in buffer (deionized H_2_O, 5% Triton X-100, and 10 × TN buffer) prior to serial PMCA that consisted of 30 s of sonication followed by 29.5 min incubation (one round is 24 h)^15,19,49^. Four hamster synthetic prions (HSP) were generated using either hamster WT or mutated recombinant PrP. The mouse and hamster PrP amino acid sequences differ at 12 residues. Analysis of which of these residues has the greatest effect on the mouse/hamster species barrier, assessed by changes in PMCA conversion efficiency, determined residues 54, 139, and 205 (hamster numbering) have the greatest impact. The mutations to the hamster recombinant PrP amino acid sequence are as follows: (1) deletion of glycine at residue 54 (HSP^ΔG54^); (2) deletion of glycine at residue 54 and substitution of methionine with isoleucine at residue 139 (HSP^ΔG54/M139I^); (3) substitution of methionine with isoleucine at residue 139 and a substitution of isoleucine with methionine at residue 205 (HSP^M139I/I205M^) (Supplemental Fig. S1). These mutations to the hamster PrP amino acid sequence increased the similarity of the hamster sequence to the murine sequence. The hamster synthetic prions were generated in PMCA using the same buffer and cofactors (RNA and POPG) as the de novo generated MSP, but were serially converted by seeded conversion. The MSP seeded conversion of hamster double mutant recombinant PrP, double mutant HSP seeded conversion of hamster single mutant recombinant PrP, and single mutant HSP seeded conversion of hamster WT recombinant PrP.

### Animal bioassay

Groups of male, 3–4 week Syrian hamsters (n = 5 per group) were inoculated with 25 μl of murine or hamster synthetic prions^15,19^ or a 10% (wt/vol) brain or spinal cord homogenate by the intracranial (i.c.) inoculation route as previously described^50^. Spinal cord homogenate was used as inoculum for third passage as whole brains were collected for histology at second passage. Hamsters were monitored three times per week for onset of clinical signs of prion disease. Incubation period was calculated as the number of days between inoculation and onset of clinical signs of prion infection. Clinical duration of disease was calculated as the number of days between onset of clinical signs and sacrifice. Animals were weighed once per week. Hamsters were considered moribund when weight declined for more than three weeks in a row or hamsters lost greater than 10 g in 1 week.

### Tissue collection and processing

Moribund animals were anesthetized with isoflurane (Patterson Veterinary, Loveland, CO) and perfused transcardially with Dulbecco’s phosphate-buffered saline (DPBS; Corning, Manassas, VA). Following euthanasia, tissues were collected using strain-dedicated tools that were decontaminated by immersion in bleach (neat) for 15 min at room temperature. Brains were collected whole for histology, collecting spinal cord (C1–C3) for biochemistry. Tissue collected for biochemical testing was immediately placed on dry ice and then stored at − 80 °C. Before use in analysis, CNS tissue was homogenized to 10% w/v (100 μg/μl) in Dulbecco’s Phosphate Buffered Saline (DPBS) (Corning, Manassas, VA) and stored at − 80 °C. Tissue collected for histological purposes was immersion fixed with paraformaldehyde-lysine-periodate (PLP) for 24 h at RT, placed in cassettes, and then stored in 70% ethanol until paraffin processing with a Tissue-Tek VIP 6 vacuum infiltration processor (Sakura Finetek USA, Torrance, CA). Thin (7 μm) sections of tissue for histology and immunohistochemistry were mounted on 25 × 75 Superfrost Plus glass slides (Fisher Scientific, Pittsburg, PA) and dried for 48 h at 37 °C.

### SDS-PAGE and western blot

Detection of PrP^Sc^ by Western blot was performed as previously described^26^. Briefly, 5% w/v brain homogenate was incubated with proteinase K (PK; 100 μg/mL stock; Roche Diagnostics, Mannheim, Germany) for 1 h at 37 °C with shaking. To halt PK digestion, an equal volume of 2 × sample buffer (4% w/v SDS, 2% v/v β-mercaptoethanol, 40% v/v glycerol, 0.004% w/v Bromophenol blue, and 0.5 M Tris buffer pH 6.8) was added and the samples were boiled at 100 °C for 10 min. Samples were size fractionated on 4–12% Bis–Tris NuPage polyacrylamide gel (Invitrogen, Carlsbad, CA), and transferred to a polyvinylidene difluoride (PVDF) membrane (Immobilon P; Millipore Sigma, MS). The membrane was blocked with 5% w/v nonfat dry milk in 0.05% v/v tween tris-buffered saline (TTBS) (BioRad Laboratories, Hercules, CA) for 30 min and the hamster prion protein detected by the mouse monoclonal anti-PrP antibody 3F4^51^ (final concentration of 0.1 μg/mL, EMD Millipore, Billerica, MA). Western blots were developed using Pierce SuperSignal West Femto maximum-sensitivity substrate per manufacturer’s instructions (Pierce, Rockford, IL) and imaged on a Li-Cor Odyssey Fc Imager (Li-Cor, Lincoln, NE). Migration analysis of the unglycosylated PrP^Sc^ polypeptide was determined using NIH ImageJ Fiji (NIH, USA) lane analysis software.

### Conformational stability assay

The PrP^Sc^ conformational stability assay was performed as described previously^26,52^. Briefly, a guanidine hydrochloride (Gdn-HCl) serial dilution was prepared by diluting 8 M Gdn-HCl (Sigma-Aldrich, St. Louis, MO) into DPBS (Corning, Manassas, VA) to final concentrations of Gdn-HCl ranging from 0 to 3.5 M (increasing by 0.5 M increments). Brain homogenate was diluted 1:10 (spinal cord homogenate diluted 1:5) into DPBS (Corning, Manassas, VA) from a 10% w/v brain or spinal cord homogenate and subsequently incubated in Gdn-HCl (1:3 diluted homogenate:Gdn-HCl dilution) with shaking for one hour at room temperature. The concentration of Gdn-HCl was adjusted to 0.5 M for all samples prior to plating onto a 96-well filter plate with a PVDF membrane bottom (Telling; Merck Millipore, Co. Cork, Ireland). Once samples were spun down and bound to the plate membrane, samples were digested with PK (5 μg/mL; 1:100 PK:BH) for one hour at 37 °C (5 μg/ml; Roche Diagnostics, Mannheim, Germany), followed by incubation with phenylmethane sulfonyl fluoride (PMSF; MP Biomedicals, LLC, Salon, OH) for 20 min at room temperature to halt PK digestion. The samples were blocked for endogenous peroxidases (0.3% H_2_O_2_ in methanol) and non-specific binding (5% w/v nonfat dry milk in TTBS (BioRad Laboratories, Hercules, CA). Hamster prion protein was detected using the mouse monoclonal anti-PrP antibody 3F4 (final concentration of 0.1 μg/mL; EMD Millipore, Billerica, MA). The membrane was developed using the Pierce SuperSignal West Femto system (Pierce, Rockford, IL) and imaged on a Li-Cor Odyssey Fc Imager (Li-Cor, Lincoln, NE). Signal intensity was analyzed using Li-cor Image Studio Software v.1.0.36 (Lincoln, NE) and denaturation curves were generated using GraphPad Prism (GraphPad Software, San Diego, CA). The point where half of PrP^Sc^ is in the native folded state and half is in a denatured state (i.e. [Gdn-HCl]_1/2_) was determined by calculating the log IC_50_ of the non-linear curve fitted to the normalized data (GraphPad Software, San Diego, CA). Only data from curves with a r^2^ value of over 0.90 were included in our analysis.

### Neuropathology analysis

Tissue analyzed for neuropathology underwent staining with hematoxylin and eosin as previously described^26,28^. Briefly, brain sections first were exposed to xylene (Fisher Scientific, Pittsburg, PA), rehydrated using an alcohol series (100–70% vol/vol ethanol; Decon Labs Inc., King of Prussia, PA), and rinsed in water. Slides then were stained with hematoxylin (Thermo Fisher Scientific, Waltham, MA) and rinsed in water. These steps (exposure to reagent followed by water rinse) were repeated with clarifier (Thermo Fisher Scientific, Waltham, MA) and bluing reagent (Thermo Fisher Scientific, Waltham, MA). Slides were then counterstained with eosin (95% ethanal (Decon Labs Inc., King of Prussia, PA), Eosin Y (Sigma-Aldrich, St. Louis, MO), Phloxine B (Sigma-Aldrich, St. Louis, MO), glacial acetic acid (Fisher Scientific, Pittsburg, PA)), dehydrated using an alcohol series (80–100% ethanol; Decon Labs Inc., King of Prussia, PA), and rinsed in xylene prior to cover slipping (Slip-Rite cover glass, 24 × 50, Fisher Scientific, Pittsburg, PA). Images of brain sections were captured using an Infinity 2 microscope camera (Teledyne Lumenera, Ottawa, ON) attached to a Nikon Eclipse 80i compound microscope (Nikon Instruments, Melville, NY) and ImageJ software.

### Immunohistochemistry

Immunohistochemistry (IHC) was performed as previously described^26,28^. Briefly, brain sections were deparaffinized and incubated in formic acid (Sigma-Aldrich, St. Louis, MO) for 10 min. To block endogenous peroxidases, slides were incubated in 0.3% v/v H_2_O_2_ in methanol for 20 min at room temperature. To block non-specific binding, sections were incubated in 10% vol/vol normal horse (or goat) serum (Vector, Burlingame, CA) in TTBS for 30 min at room temperature. Sections were incubated with either the monoclonal anti-PrP antibody 3F4^51^ (final concentration of 3.33 μg/mL; EMD Millipore, Billerica, MA), anti-glial fibrillary acidic protein antibody (GFAP; final concentration of 1.45 μg/mL; Abcam, Cambridge, MA), or anti-Iba1 antibody (final concentration of 0.67 μg/mL; DakoCytomation, Glostrup, Denmark) overnight at 4 °C. Sections were next incubated with either horse or goat anti-mouse biotinylated antibody (1:700; Vector, Burlingame, CA) for 30 min at room temperature followed by ABC solution (Vector, Burlingame, CA) for 20 min at room temperature. The chromogen was developed with 0.05% w/v DAB (3,3′-Diaminobenzidine) in tris-buffered saline (TBS) with 0.003% v/v or 0.0015% v/v H_2_O_2_ in MilliQ water and counterstained with hematoxylin. Images of brain sections were captured as described above.

### Equipment and settings

For Figs. 1 and 3a, Western blots were developed using Pierce SuperSignal West Femto maximum-sensitivity substrate per manufacturer’s instructions (Pierce, Rockford, IL) and imaged on a Li-Cor Odyssey Fc Imager (Li-Cor, Lincoln, NE) using the chemiluminescence channel. Exposure was increased to visualize low levels of PrP^Sc^ and the unglycosylated bands for Figs. 1 and 3a, respectively. For Fig. 5, images of brain sections were captured using an Infinity 2 microscope camera (Teledyne Lumenera, Ottawa, ON) attached to a Nikon Eclipse 80i compound microscope (Nikon Instruments, Melville, NY) and ImageJ software. Main images were captured at 10 × magnification and inset images were captured at 40 × magnification. Dimensions of all images at capture were 2448 × 2048 pixels. Images were processed identically for white balance using Adobe Lightroom (Adobe, San Jose, CA).

### Statistical analysis

Differences among groups for biochemical properties such as conformational stability was determined by one-way ANOVA (p < 0.05) using GraphPad Prism (GraphPad Software, San Diego, CA).

## Supplementary Information

Supplementary Information.

## Data Availability

All relevant data are within the manuscript and its Supporting Information files.
