# Supplementary material for: Prion protein amino acid sequence influences formation of authentic synthetic PrPSc
Source: Sci Rep. 2023 Jan 9;13:441. doi: 10.1038/s41598-022-26300-0 (PMC9829857; doi:10.1038/s41598-022-26300-0)
Supplement: Supplementary file 1 — Supplementary Information. [file 41598_2022_26300_MOESM1_ESM.docx]

**SUPPLEMENTAL INFORMATION**

**
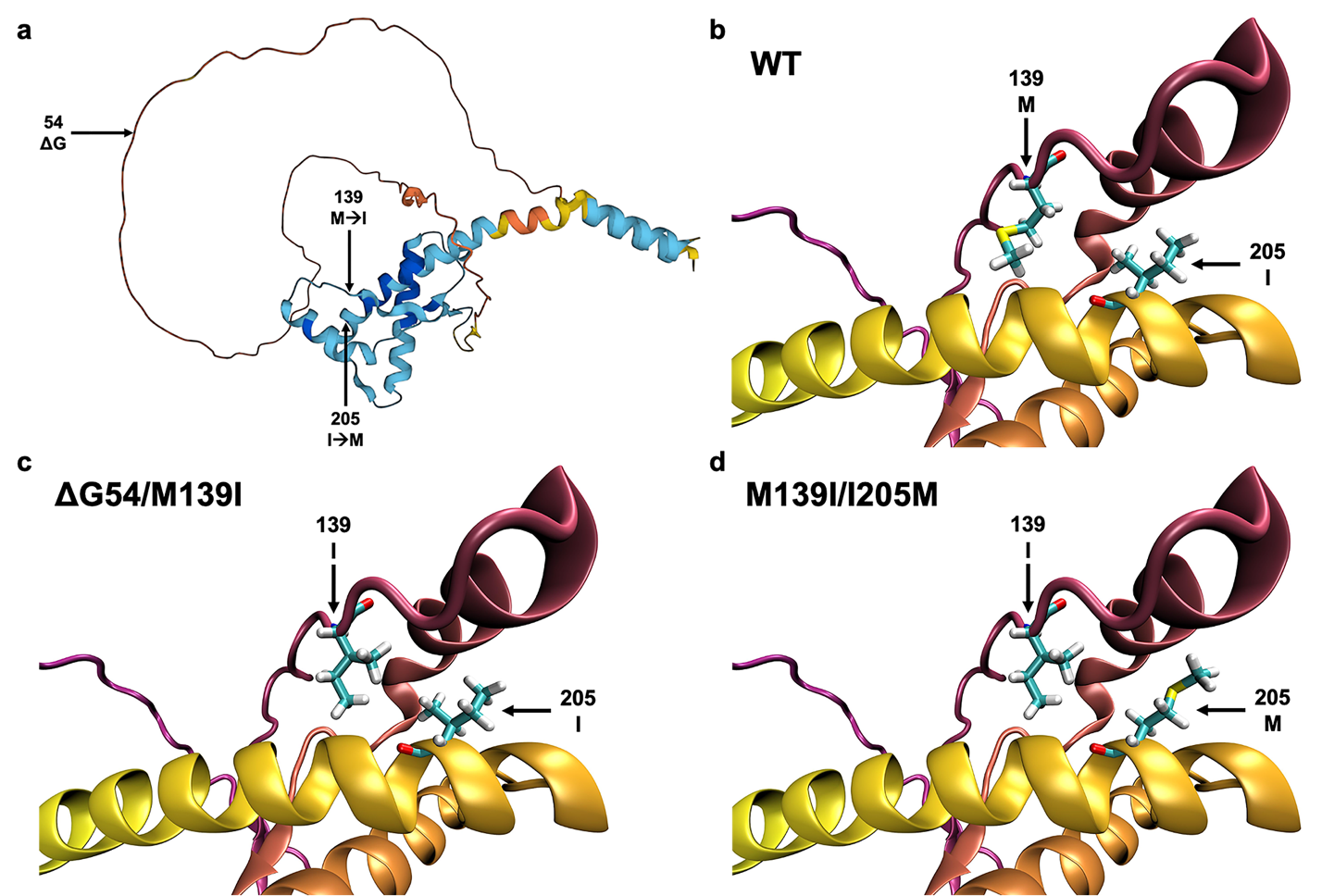
**

**Supplemental Figure S1. Location of hamster PrP mutations used to generate the hamster synthetic prions (HSP).** (a) WT hamster PrP^C^ structure highlighting locations of three mutations (residues 54, 139, and 205). (b-d) Close ups of residues 139 and 205 in hamster WT, ΔG54/M139I, and M139I/I205M PrP, respectively. The ΔG54 mutation is within the first octapeptide repeat region and the amino acid substitutions at 139 and 205 are within the β-strand region between β-sheet 1 and α-helix 1 and α-helix 3, respectively. The PrP structures in panels a-d are from the AlphaFold Protein Structure Database (UniProt ID P04273; https://www.alphafold.ebi.ac.uk)^1,2^. The structures in panels B-D were visualized using Visual Molecular Dynamics (VMD) software. Charmm-Gui PDBreader was used to mutate residues.

**
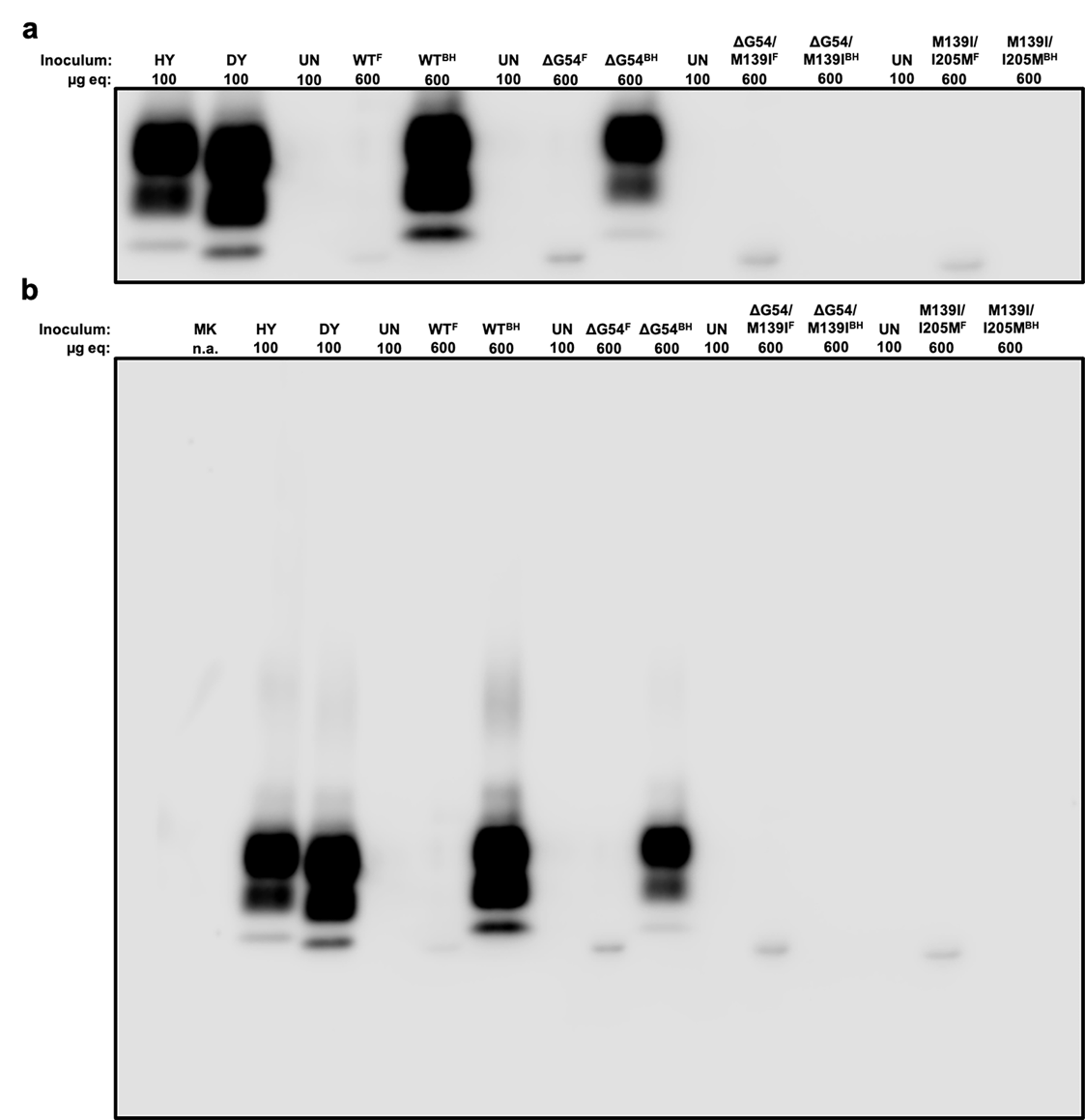
**

**Supplemental Figure S2. Western blot of HSP and 1^st^ passage HSP in hamsters.** Western blot of PK-digested HSP^WT^, HSP^ΔG54^, HSP^ΔG54/M139I^, and HSP^M139I/I205M^ synthetic prions compared to PK-digested brain homogenate from HSP^WT^-, HSP^ΔG54^-, HSP^ΔG54/M139I^-, and HSP^M139I/I205M^-infected hamsters. Western blot analysis with the anti-PrP antibody 3F4 identified the hamster synthetic prions (WT and mutants) have a PK-resistant core that appears to migrate faster than the unglycosylated bands of PrP^Sc^ from HY-, DY-, and HSP (WT and mutant)-infected brain homogenate. Once the HSP are passaged in hamsters, all three glycoforms are observed. Panel a is cropped to focus on PrP^Sc^ and panel b is the full, uncropped gel. Microgram equivalents (μg eq) loaded for each lane are listed above the blot. ^F^ – synthetic prion; ^BH^ – HSP-infected brain homogenate from 1^st^ passage in hamsters.

**
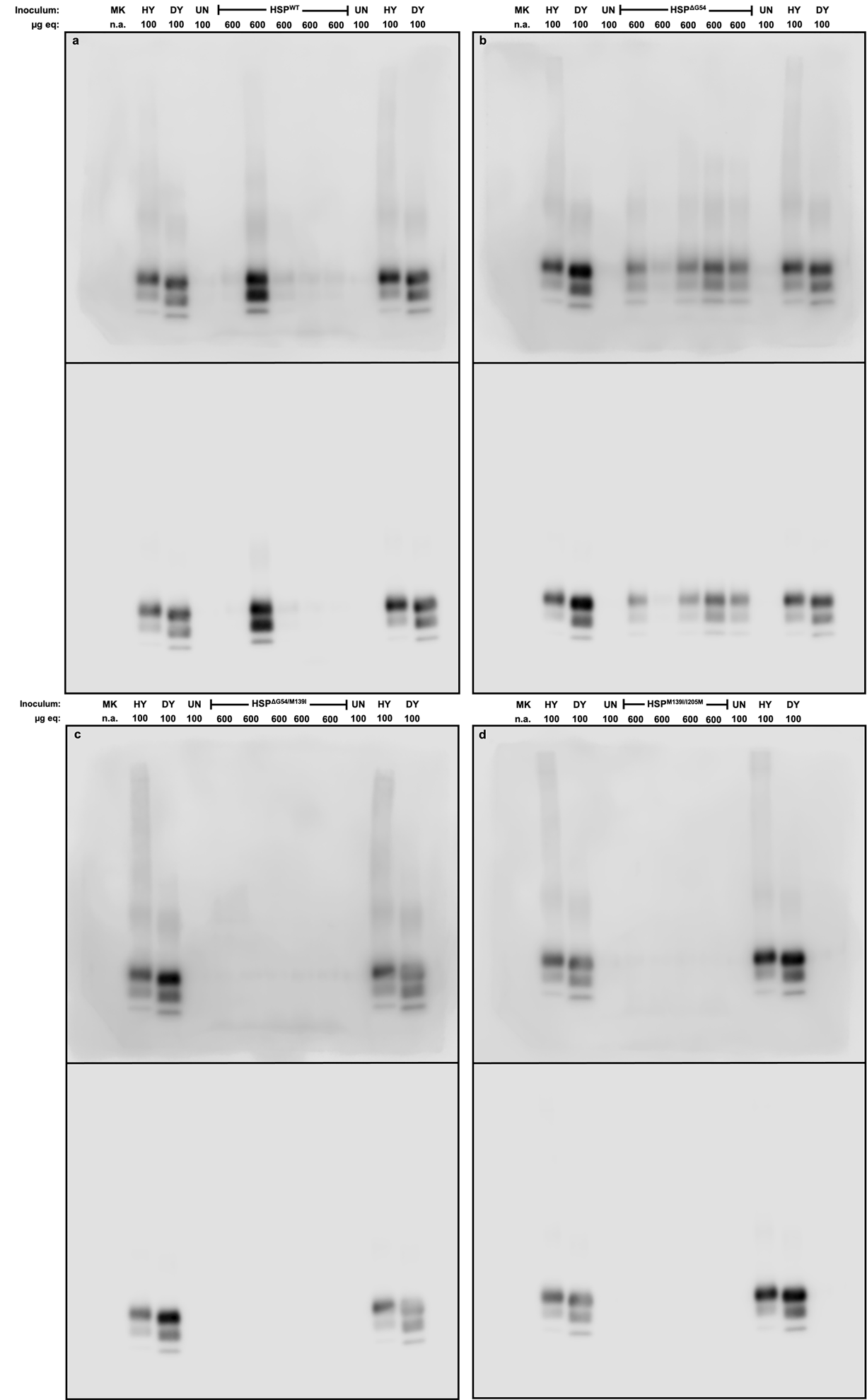
**

**Supplemental Figure S3. Uncropped Western blots of Figure 1.** Representative Western blots of brain homogenate from (a) HSP^WT^-, (b) HSP^ΔG54^-, (c) HSP^ΔG54/M139I^-, or (d) HSP^M139I/I205M^-infected hamsters. The top portion in each panel is darkened to show the membrane edges and the bottom portion is the original untouched image. Uninfected (UN), HY, and DY Western blot controls on the right side were cropped for Figure 1. Microgram equivalents (μg eq) loaded for each lane are listed above each gel.

**
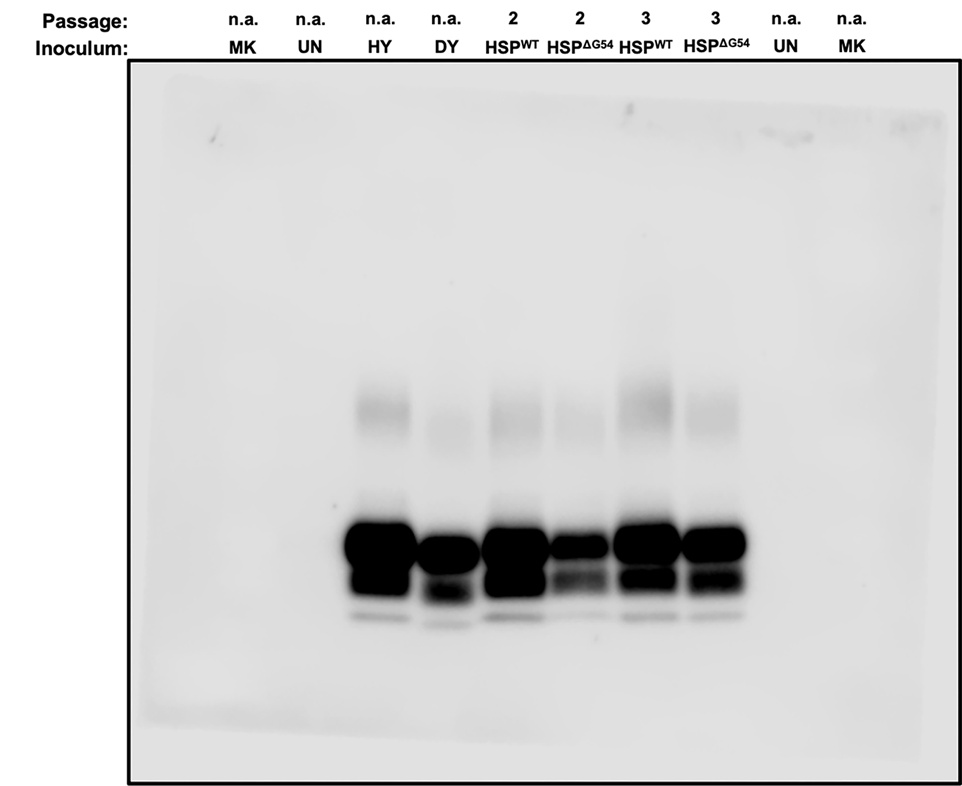
**

**Supplemental Figure S4. Uncropped Western blot for Figure 3a.** Western blot of PrP^Sc^ from the CNS of hamsters inoculated with HY, DY, or second or third hamster passage of hamster synthetic prions (HaHSP^WT^ or HaHSP^ΔG54^). The UN control and marker (MK) on the right and the MK on the left were cropped for Figure 3a.

**SUPPLEMENTAL REFERENCES**

1 Jumper, J. *et al.* Highly accurate protein structure prediction with AlphaFold. *Nature* **596**, 583-589, doi:10.1038/s41586-021-03819-2 (2021).

2 Varadi, M. *et al.* AlphaFold Protein Structure Database: massively expanding the structural coverage of protein-sequence space with high-accuracy models. *Nucleic Acids Res* **50**, D439-D444, doi:10.1093/nar/gkab1061 (2022).
